# The impact of harmonization on radiomic features in Parkinson’s disease and healthy controls: A multicenter study

**DOI:** 10.3389/fnins.2022.1012287

**Published:** 2022-10-10

**Authors:** Benedetta Tafuri, Angela Lombardi, Salvatore Nigro, Daniele Urso, Alfonso Monaco, Ester Pantaleo, Domenico Diacono, Roberto De Blasi, Roberto Bellotti, Sabina Tangaro, Giancarlo Logroscino

**Affiliations:** ^1^Dipartimento di Ricerca Clinica in Neurologia, Centro per le Malattie Neurodegenerative e l’Invecchiamento Cerebrale, Pia Fondazione Cardinale G. Panico, Università degli Studi di Bari Aldo Moro, Lecce, Italy; ^2^Dipartimento di Scienze Mediche di Base, Neuroscienze e Organi di Senso, Università degli Studi di Bari Aldo Moro, Bari, Italy; ^3^Istituto Nazionale di Fisica Nucleare (INFN), Sezione di Bari, Bari, Italy; ^4^Dipartimento Interateneo di Fisica M. Merlin, Università degli Studi di Bari Aldo Moro, Bari, Italy; ^5^Istituto di Nanotecnologia, Consiglio Nazionale delle Ricerche (CNR-NANOTEC), Lecce, Italy; ^6^Department of Neurosciences, King’s College London, Institute of Psychiatry, Psychology and Neuroscience, London, United Kingdom; ^7^Dipartimento di Radiologia, Pia Fondazione Cardinale G. Panico, Lecce, Italy; ^8^Dipartimento di Scienze del Suolo, Della Pianta e degli Alimenti, Università degli Studi di Bari Aldo Moro, Bari, Italy

**Keywords:** radiomics analysis, ComBat, multi-site harmonization, structural MRI, Parkinson’s disease

## Abstract

Radiomics is a challenging development area in imaging field that is greatly capturing interest of radiologists and neuroscientists. However, radiomics features show a strong non-biological variability determined by different facilities and imaging protocols, limiting the reproducibility and generalizability of analysis frameworks. Our study aimed to investigate the usefulness of harmonization to reduce site-effects on radiomics features over specific brain regions. We selected T1-weighted magnetic resonance imaging (MRI) by using the MRI dataset *Parkinson’s Progression Markers Initiative* (PPMI) from different sites with healthy controls (HC) and Parkinson’s disease (PD) patients. First, the investigation of radiomics measure discrepancies were assessed on healthy brain regions-of-interest (ROIs) *via* a classification pipeline based on LASSO feature selection and support vector machine (SVM) model. Then, a ComBat-based harmonization approach was applied to correct site-effects. Finally, a validation step on PD subjects evaluated diagnostic accuracy before and after harmonization of radiomics data. Results on healthy subjects demonstrated a dependence from site-effects that could be corrected with ComBat harmonization. LASSO regressor after harmonization was unable to select any feature to distinguish controls by site. Moreover, harmonized radiomics features achieved an area under the receiving operating characteristic curve (AUC) of 0.77 (compared to AUC of 0.71 for raw radiomics measures) in distinguish Parkinson’s patients from HC. We found a not-negligible site-effect studying radiomics of HC pre- and post-harmonization of features. Our validation study on PD patients demonstrated a significant influence of non-biological noise source in diagnostic performances. Finally, harmonization of multicenter radiomic data represent a necessary step to make analysis pipelines reliable and replicable for multisite neuroimaging studies.

## Introduction

Radiomics is a challenging development area in imaging field that is greatly capturing interest of radiologists and neuroscientists (Kumar et al., 2012; Gillies et al., 2016; Salvatore et al., 2019; Guiot et al., 2022). Allowing quantitative radiographic phenotyping over several types of magnetic resonance imaging (MRI) acquisition, radiomic analysis has been proposed as a primary task to improve knowledge about diagnosis, prognosis and predictions of pharmaceutical response in oncology and neurodegenerative diseases (Mayerhoefer et al., 2020). Moreover, thanks to its capability to extract engineered measures from specific regions of interest (ROIs), radiomics has shown to be a useful approach for characterizing and classifying patients with pathological conditions (Gillies et al., 2016; Feng and Ding, 2020). Indeed, many oncological applications have demonstrated the radiomics ability to capture intra-tumoral heterogeneity in a non-invasive way. Concerning neurodegenerative diseases, instead, recent studies on Alzheimer’s (AD) and Parkinson’s diseases (PD) have highlighted the potentiality of radiomics to detect abnormalities beyond standard morphological imaging markers. In particular, radiomics approach had achieved interesting results in distinguishing patients with PD from controls (Cao et al., 2020; Liu et al., 2020) and from atypical parkinsonian syndromes (Tupe-Waghmare et al., 2021). Moreover, associations between radiomics measures and clinical variables have been described in both cross-sectional and longitudinal studies (Feng et al., 2018; Salmanpour et al., 2022).

Despite the outstanding results, radiomics features have showed a strong dependence from different research facilities or different acquisition protocols, limiting the reproducibility and generalizability of the proposed frameworks especially for application on multi-site dataset (Nieuwenhuis et al., 2017). Recent studies have addressed this issue using an intensity normalization step before the feature extraction (Nyul et al., 2000; Shinohara et al., 2014; Reinhold et al., 2018; Dewey et al., 2019). However, the elimination of non-biological variability caused by site-effects represents a not-trivial problem that makes sometimes the normalization approach ineffective for application on multi-scanner datasets (Eshaghzadeh Torbati et al., 2021; Li et al., 2021). Therefore, latest applications in the field of oncology have proposed an additional step of feature harmonization based on ComBat method (Crombé et al., 2020; Da-Ano et al., 2020; Li et al., 2021; Mali et al., 2021), originally implemented as batch-effect correction method for microarray expression data (Johnson et al., 2007). This approach has been also applied to classical morphometric properties such as cortical thickness, cortical surface area and subcortical volumes in brain MRI removing scan effect and increasing the power and statistical significance of the results (Fortin et al., 2018; Pomponio et al., 2020; Radua et al., 2020; Eshaghzadeh Torbati et al., 2021).

In the current study, we investigated the effectiveness of normalization and harmonization approaches to reduce site-effects on radiomics features from healthy brain ROIs. At first, we extracted radiomics features on T1-weighted MRI images of healthy subjects collected from different acquisition sites in the context of the *Parkinson’s Progression Markers Initiative* (PPMI), sponsored by the *Michael J. Fox Foundation*, evaluating the sensitivity to site-related effects. In a second step, normalization and harmonization models defined on healthy subjects were applied on patients with PD to evaluate the classification performance pre- and post- site-effect correction.

## Materials and methods

### Participants

Data used in the preparation of this study were obtained from the PPMI database.^1^ For up-to-date information on the study, visit ppmi-info.org. The T1-weighted MR images selected for this study were acquired using a 1.5–3 Tesla scanner from different manufactures (Philips, GE, Siemens). Acquisition protocols from each site are reported in Supplementary Table 1.

### Magnetic resonance imaging pre-processing

Structural MR images were segmented using the *recon-all* script included in Freesurfer v6.0.^2^ After removal of non-brain tissue and bias of each structural brain image, we used the non-uniform intensity corrected image (*nu.mgz*) in the Freesurfer space to compute radiomics features. An additional step of intensity normalization was performed using the *Z-Score* method by centering each pre-processed T1w volume at the mean with standard deviation. To identify and characterize the site effect on radiomic features, we extracted the ROIs using the Desikan–Killiany atlas cortical parcelation from the individual subcortical segmentation image (*aparc* + *aseg.mgz*) (Desikan et al., 2006). Then, we thresholded each brain parcelation using FSL (Smith et al., 2004) tools to extract the binary masks for the next radiomics analysis.

### Radiomics features extraction and harmonization

Data used for our analysis was collected from three ROIs, namely Caudate, Putamen and Thalamus, for both hemispheres, as a set of subcortical brain regions pertinent to PD (Shimohama et al., 2003; Halliday, 2009). For each ROI, we defined a set of 88 radiomic features, including 18 first-order features to describe voxel intensity distribution within image mask, and 70 second-level textural measures to highlight spatial distribution of voxels through four different matrices: 24 features from Gray Level Co-occurrence Matrices (GLCM), 16 from Gray Level Run Length Matrices (GLRLM), 14 measures from Gray Level Dependence Matrices (GLDM) and 16 features from Gray Level Size Zone Matrices (GLSZM) (detailed information about extracted features are reported in Supplementary Table 2; Zwanenburg et al., 2020). The extraction procedure was implemented using Pyradiomics, an open-source Python package (Van Griethuysen et al., 2017).

The multicenter harmonization was performed using ComBat algorithm (Johnson et al., 2007) for location (mean) and scale (variance) adjustments of data due to the site differences between subjects. Particularly, this approach assume that the batch effects can be modeled out by standardizing means and variances across batches. We applied the generalized additive model (GAM) of ComBat, also called NeuroHarmonize, that considered sex and non-linear age effects as covariates in the input data (Pomponio et al., 2020). More in details, this method combines the ComBat harmonization pipeline (Fortin et al., 2017, 2018), with the GAM (Hastie and Tibshirani, 1986). The former aims to remove unwanted sources of variability due to site differences, while preserving the variability due to other biological significant covariates; the latter introduces a penalized non-linear term to better take into account the age effects and capture also non-linearities in age-related differences in radiomic feature. In contrast to a general linear model approach that includes site as a fixed effect covariate, the GAM of ComBat considers only age and sex as covariates to control for during harmonization. This approach assumes that for a given site, the effects across features derive from a common distribution, and thus borrows information across features to shrink estimates toward a common mean. In addition to removing additive site effects, ComBat also corrects multiplicative site effects by removing heteroscedasticity of model errors across site. In our framework, NeuroHarmonize was implemented using Empirical Bayes framework, which is useful for harmonizing multiple features, such as brain regional measures. The estimation of the site hyperparameters (γ as an additive batch effect affecting the measurement, δ as a multiplicative batch effect) of the prior distribution for site-effect correction was conducted considering only healthy controls (HC). Of note, to ensure unbiased results, the harmonization parameters was calculated over control subjects in the training set of each cross-validation fold and then applied on the remaining subjects in train and test folds to correct the site-effect. The Python implementation of harmonization framework was found at https://github.com/rpomponio/neuroHarmonize (Pomponio et al., 2020).

### Radiomics modeling

To characterize site effects on radiomics features, a “site vs. site” classification model was built in Leave-One-Out cross validation (LOOCV) considering only HC. To this end, classification performances were first evaluated using raw data. Next, we evaluated the impact of image normalization and ComBat harmonization on the classification performances. For each classification model, at each inner loop of LOOCV, we firstly reduced the burden of high dimensionality of radiomics set of features using least absolute shrinkage and selection operator (LASSO) (Tibshirani, 1996; Friedman et al., 2010). Therefore, the optimal penalty parameter of LASSO was defined *via* minimization of “Binomial Deviance” and features with non-zero regression coefficients were retained. Then, for classification purpose, we trained a radial basis Support Vector Machines (SVM) model (Cortes and Vapnik, 1995; Chang and Lin, 2011) for each binary site-classifier on previously selected radiomics features.

**FIGURE 1:**
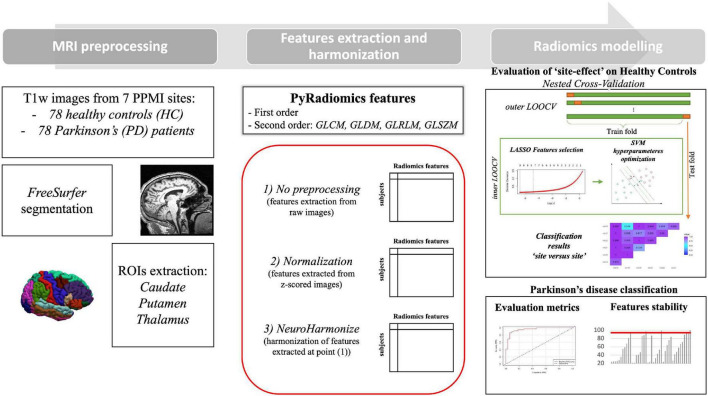
Processing pipeline.

As second step of our framework, we implemented the same pipeline for PD detection. This procedure was implemented in a ten-times repeated 10-fold cross validation setting. At each of 100 bootstraps, nine folds was used to define a LASSO regression model to select optimal radiomics features. The selected features were saved in a vector to further analysis (Lombardi et al., 2020). These features were also used to train a radial basis SVM. At each iteration, we tested the predictive power of the model by using the excluded fold. The whole pipeline is illustrated in Figure 1.

### Statistical analysis

Demographic and clinical information of the dataset were provided with descriptive statistics (mean ± SD). Group differences in age, sex, MoCA (Montreal Cognitive Assessment), UPDRS-III (Unified Parkinson’s Disease Rating Scale) scales and H&Y (Hoehn and Yahr) stage were investigated through Chi-square test, one-way analysis of variance (ANOVA) and Kruskal–Wallis ANOVA followed by *post-hoc* comparisons. For all analyses, the corrected significance threshold was set at *p* < 0.05 after Bonferroni’s correction for multiple comparisons. Statistical analysis was performed by using R software (Version 3.6.3: R Foundation for Statistical Computing, Vienna, Austria).

The Area Under the receiving operating characteristic Curves (AUCs) were used as evaluation metric for our “site vs. site” models. Classification performances for HC vs. PD models were evaluated by accuracy, sensitivity and specificity, mediated over the 100 bootstraps of classification. Finally, the diagnostic capabilities of the radiomics signatures were evaluated with Receiver Operating Characteristic (ROC) curve analysis.

To assess the stability of radiomics features selected by LASSO regression over HC vs. PD model, we used a frequency-based criterion. For each round of the bootstraps, we stored as relevant features only those corresponding to non-zero weights assigned by LASSO. Subsequently, we selected as most stable radiomics features those that occurred in at least 95° percentile of the frequency vector.

## Results

### Demographic and clinical data

We selected MR images from seven sites of the PPMI database according with the number of enrolled HC. Table 1 reports all demographic and clinical details for each clinical site included in our study. No statistical difference was found between HC from clinical sites in age, sex, H&Y score and MoCA scales.

**TABLE 1:** Demographic and clinical details of healthy controls for each site.

	Site 12	Site 19	Site 20	Site 21	Site 22	Site 27	Site 52	*P*-value
n	10	12	12	11	11	10	11	
Age [mean (SD)]	58.56 (13.68)	52.73 (13.81)	58.08 (10.37)	60.47 (8.75)	63.16 (11.86)	58.98 (7.63)	64.25 (6.55)	–
Female (%)	5 (50.0)	4 (33.3)	6 (50.0)	4 (36.4)	2 (18.2)	2 (20.0)	7 (63.6)	–
Hoehn and Yahr stage = 0 (%)	10 (100.0)	12 (100.0)	12 (100.0)	11 (100.0)	11 (100.0)	10 (100.0)	11 (100.0)	–
MoCA [mean (SD)]	28.30 (0.82)	28.92 (1.16)	28.25 (0.97)	27.82 (0.87)	28.55 (1.13)	28.60 (1.17)	27.82 (0.98)	–

For each site, we also selected a set of age- and sex-matched PD. Data is reported in Table 2. As expected, we found significant differences in H&Y score, UPDRS-III and MoCA scales (*p*-values < 0.001) between HC and PD patients.

**TABLE 2:** Demographic and clinical details of patients with Parkinson’s disease and healthy controls.

	PD	HC	*P*-value
N	78	78	
Age [mean (SD)]	58.85 (9.68)	59.37 (10.93)	–
Female (%)	28 (36.4)	30 (39.0)	–
Hoehn and Yahr stage (%)			–
0	0 (0.0)	77 (100.0)	
1	41 (53.2)	0 (0.0)	
2	36 (46.8)	0 (0.0)	
UPDRS-III [mean (SD)]	20.01 (9.06)	1.10 (1.95)	<0.001
MoCA [mean (SD)]	27.16 (2.42)	28.32 (1.06)	<0.001

### Characterization and correction of the “Site Effects” on healthy controls

**FIGURE 2:**
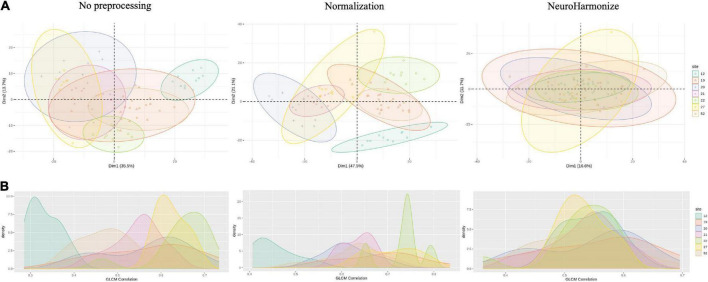
**(A)** Plots of the first 2 principal components (*dim*) from principal component analysis (PCA), colored by site; **(B)** histograms of example radiomic feature for raw, normalized, and harmonized processing.

**FIGURE 3:**
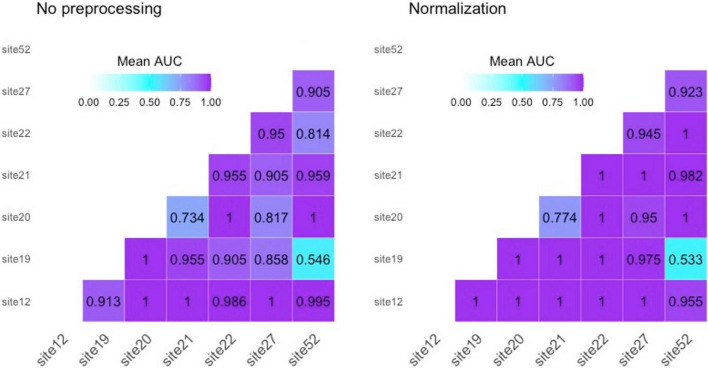
Area under the receiving operating characteristic curves (AUCs) for “site vs. site” classification on healthy controls. **Left panel** reports performances for not pre-processed radiomics features; **right panel** reports results using radiomics features from normalized MRI.

As first step, we studied the variability of radiomics features from healthy ROIs in different acquisition sites. Figure 2 presents, respectively, bivariate scatter plots of the first two principal components (*dim*) from a principal component analysis (PCA) (Figure 2A) and a histogram-based representation on one textural feature (i.e., GLCM-Correlation) (Figure 2B) for no pre-processed/normalized/harmonized approaches. As expected, a large proportion of the variation by site was corrected by harmonization of data. In Figure 3, we also report AUCs results from each binary comparison across HC from different sites. Classification performance of raw radiomics features (Figure 3, left panel) showed optimal discriminative power for all comparisons, except for *Site 19* vs. *Site 52*. Similar results were obtained using radiomics features calculated from normalized MRI (Figure 3, right panel). To evaluate pairwise differences between the models, we also performed a Wilcoxon signed rank test obtaining a *p*-value of 0.035.

After harmonization of radiomics features, LASSO regressions were unable to select any features for prediction of the outcomes. Indeed, LOOCV plots for each pairwise site comparison resulted in a penalty factor that shrinked all regression coefficients to zero (see Supplementary Figure 1).

### Parkinson’s disease classification

**FIGURE 4:**
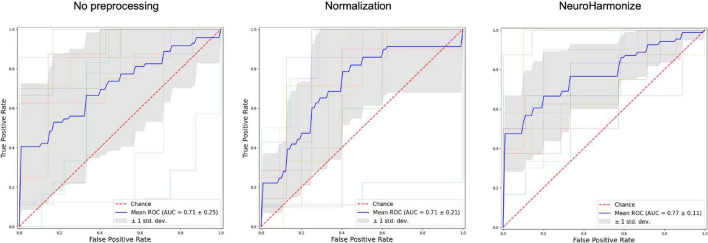
Receiver operating characteristic (ROC) curves for three different radiomics features processing.

As second step of our analysis, we evaluated goodness of classification of PD from HC in our three different radiomics approaches, namely without any pre-processing prior to radiomics features calculation, with normalization of image before radiomics computations and with harmonization from site-effect of radiomics features. We report results of each model in Table 3. Figure 4 also shows the corresponding ROCs for trained SVM. Respect to raw and normalized radiomics implementations, harmonization of features determined an increased classification power of the radiomics model.

**TABLE 3:** Performances of PD classification model from different pre-processed radiomics features.

	Accuracy (mean + st .dev)	Sensitivity (mean + st. dev)	Specificity (mean + st.dev)	AUC (mean + st.dev)
No pre-processing	0.700 + 0.120	0.730 + 0.140	0.669 + 0.182	0.709 + 0.247
Normalization	0.713 + 0.078	0.811 + 0.115	0.662 + 0.134	0.715 + 0.212
NeuroHarmonize	0.710 + 0.122	0.754 + 0.173	0.685 + 0.161	0.766 + 0.110

**FIGURE 5:**
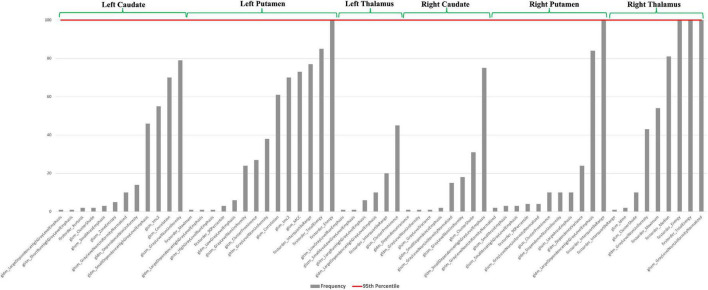
Frequency of radiomics features for PD vs. HC classification. Red line defines 95th percentile beyond which we choose the most stable measures.

As shown in Figure 5, we found five features over 95th percentile as most stable, with a predominance of radiomics measures in the right thalamus, involving energy features as measure of the magnitude of voxel values in an image, and “Gray Level Non-Uniformity Normalized” measure, quantifying the variability of gray-level intensity values in the image. Moreover, we found features in putamen, bilaterally, as most frequent predictors.

## Discussion

This study demonstrated the sensitivity of radiomic features to site-effects in multicenter neuroimaging study. We firstly investigated the problem of data variability due to non-biological effects on healthy brain regions. Then, implementing a ComBat-based harmonization procedure of radiomics features, we modelized site-related noise source reducing differences across healthy subjects. Lastly, as validation task, we evaluated the effectiveness of our harmonization approach for classification of PD patients.

The small sample size made necessary some methodological choices. First of all, it was mandatory to use a feature selection method to avoid a course of dimensionality problem due to the imbalance between the number of radiomic features and the sample size (Koutroumbas and Theodoridis, 2008; Zollanvari et al., 2020). On the other hand, a feature selection method such as LASSO was preferred over other feature reduction methods (PCA, LDA, etc.) in order to achieve a more explainable model (Lombardi et al., 2021). Indeed, the cross-validated optimization of penalty factor for LASSO feature selection allowed to define the most important predictors over the radiomics features, also guaranteeing the interpretability of the model in the clinical/radiological field (Lombardi et al., 2022). Overall, the implementation of a leave-one-out cross-validation procedure for site-vs.-site classification (compared to k-fold cross-validation as a bias-variance tradeoff) was used to guarantee approximately unbiased results over our small sample size. Concerning site-effects on radiomic features, PCA and histogram-based representation of radiomics measures highlighted the need of correcting for site effects before performing further analyses. Indeed, this allowed to distinguish HC from each site with high accuracy. Similar results were observed for normalized images. By contrast, after application of NeuroHarmonize algorithm, no subset of features could be identified to differentiate HCs each other. On note, only comparison between subjects of *Site 19* and *Site 52* reported an AUC close to random choice without using the harmonization approach, probably due to common scanner and protocol parameters used in MRI acquisition.

These findings demonstrated an effective dependency of radiomics features from scanner and acquisition protocol that could not be eliminated with normalization of image intensity but only using a ComBat-based algorithm. Our results were in line with previous findings in radiomics, over both oncology and neuroimaging, that have demonstrated a better standardization capabilities of ComBat-based models compared with different intensity normalization techniques, such as normalization (Z-Score), WhiteStripe and Ravel (Nyul et al., 2000; Shinohara et al., 2014; Reinhold et al., 2018; Dewey et al., 2019; Eshaghzadeh Torbati et al., 2021; Li et al., 2021).

As further result, harmonization pipeline applied to PD patients allowed to improve prediction performance with respect to raw data, suggesting that site noise factor might affect classification performance in multicenter study using radiomic features. Moreover, classification performance obtained in the current study using thalamus, caudate and putamen overcame results reported in previous radiomics studies using similar region-based approach for PD classification (Liu et al., 2020; Tupe-Waghmare et al., 2021). Specifically, Tupe-Waghmare et al. (2021) achieved 0.72 of AUC highlighting the T1 radiomics of substantia nigra as most predictive features. On the other hand, Liu et al. (2020) studied radiomics of putamen and caudate separately on T2w MRI with a 0.77 of AUC of caudate model. Our study further confirmed the impact of right thalamus, besides the involvement of more classical neostriatal regions (*caudate* + *putamen*) (Sikiö et al., 2015), in PD pathophysiology, highlighting at the same time its usefulness as diagnostic marker for PD.

Our work has some limitations. Firstly, the limited sample size for each site could produce unstable LASSO regression results, as well as a possible overfitting in SVM training, that we have tried to overcome with a bootstrapped 10-fold CV. Overall, cohorts of subjects from other international neuroimaging studies can be added to solve these issues guarantying greater generalizability of the results. Second, we applied only one type of normalization and harmonization techniques limiting other possible comparisons and further optimizations of performances. Future works can consider more complex intensity normalization methods, such as RAVEL or WhiteStripe, and recently developed alternative versions of ComBat with improved flexibility (M-ComBat) and robustness (B-ComBat) (Da-Ano et al., 2020).

The harmonization of MRI data represents a crucial problem in several medical imaging applications due to non-biological effects determined by different acquisition sites, scanners and multiparametric sequences. Our study aimed to assess the variability of radiomics features extracted on T1w MR images collected in a multicentric context. We found a not-negligible site-effect comparing radiomics features of HC pre- and post-harmonization pipeline. Moreover, our study demonstrated a significant influence of scan noise in distinguishing controls from PD patients. Overall, harmonization of radiomic features represents a necessary requirement for reliable and replicable analysis frameworks in multicenter study.

## Data availability statement

Publicly available datasets were analyzed in this study. This data can be found here: https://www.ppmi-info.org/access-data-specimens/download-data.

## Ethics statement

The PPMI study was approved by the local Institutional Review Boards of all participating sites (https://www.ppmi-info.org/about-ppmi/ppmi-clinical-sites/) and written informed consent for imaging data and clinical questionnaires was obtained from each participant at the time of enrollment

## Author contributions

BT: conceptualization, formal analysis, methodology, software, visualization, and roles/writing – original draft. AL: conceptualization, methodology, validation, supervision, and writing – review and editing. SN: methodology, project administration, supervision, validation, and writing – review and editing. DU: data curation, resources, and writing – review and editing. AM, EP, and DD: data curation and writing – review and editing. RD: validation, visualization, and writing – review and editing. RB: project administration and writing – review and editing. ST: conceptualization, project administration, supervision, and writing – review and editing. GL: funding acquisition, conceptualization, project administration, supervision, and writing – review and editing. All authors contributed to the article and approved the submitted version.

## Footnotes

1
www.ppmi-info.org/access-data-specimens/download-data

2
https://surfer.nmr.mgh.harvard.edu/
