# Supplementary material for: The impact of harmonization on radiomic features in Parkinson’s disease and healthy controls: A multicenter study
Source: Front Neurosci. 2022 Oct 10;16:1012287. doi: 10.3389/fnins.2022.1012287 (PMC9589497; doi:10.3389/fnins.2022.1012287)
Supplement: Supplementary file 1 [file Data_Sheet_1.docx]

Supplementary Material

**Supplementary Figure 1.** Averaged optimization curves of LOOCV LASSO regression for each pairwise ‘Site vs Site’ model using harmonized radiomics features. The optimal penalty parameter of LASSO was defined via minimization of ‘Binomial Deviance’ and features with non-zero regression coefficients were retained. The order of magnitude of the error is such as not to allow the definition of a minimum and therefore discriminative features between sites.

**
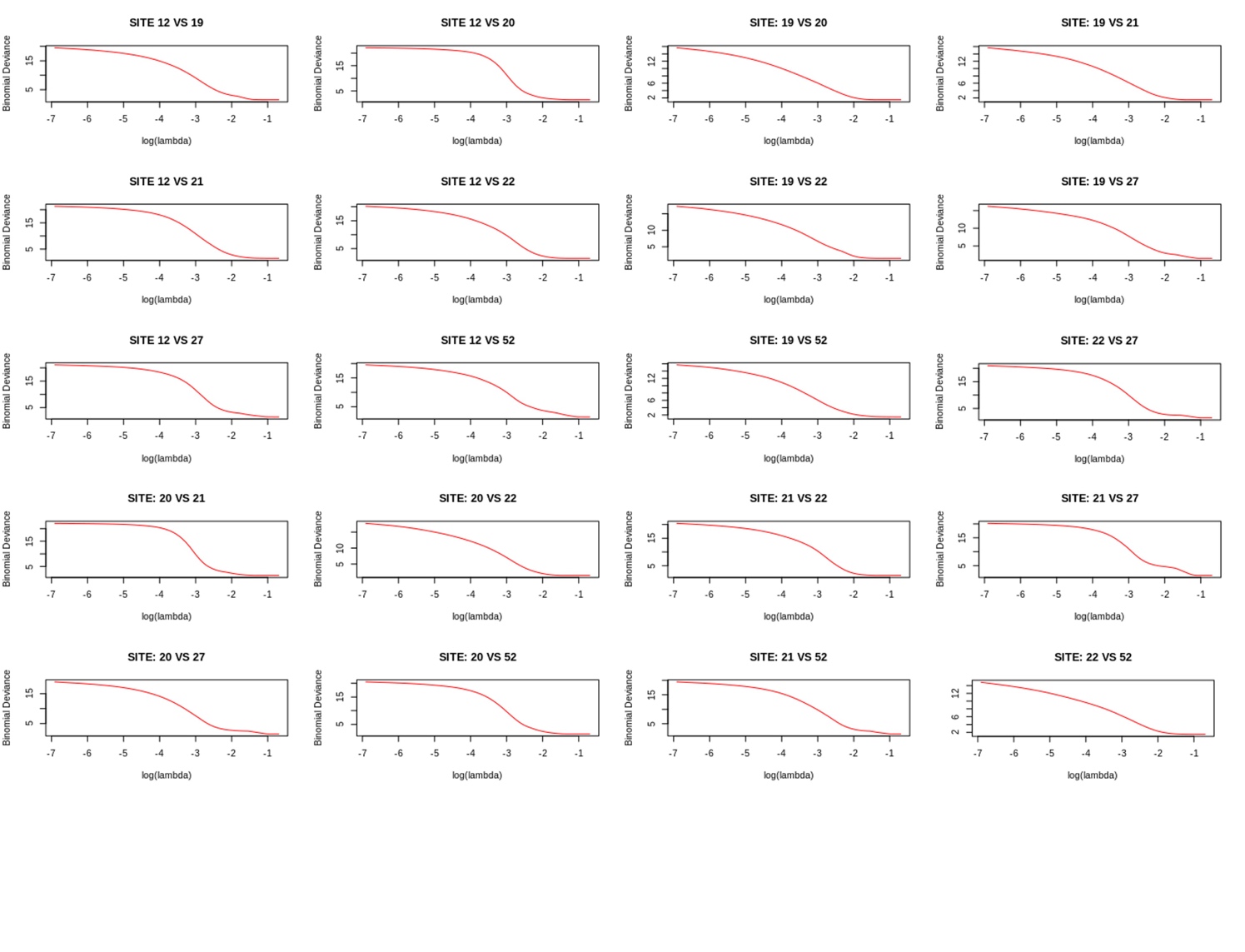
**

**Supplementary Table 1.** Description of the scanning parameters of PPMI study.

| *Site* | *ManUfacturer* | *Platform* | *Field (T)* | *TR (ms)* | *TE (ms)* | *Angle (°)* | *ST (mm)* |
| --- | --- | --- | --- | --- | --- | --- | --- |
| Site 12 | Philips Medical Systems | Achieva | 1.5 | 7.0 | 3.2 | 8 | 1.0 |
| Site 19 | Siemens | TrioTim | 3 | 2300.0 | 3.0 | 9 | 1.0 |
| Site 20 | Siemens | Symphony | 1.5 | 1980.0 | 3.9 | 15 | 2.0 |
| Site 21 | Philips Medical Systems | Gyroscan NT | 1.5 | 8.5 | 4.0 | 8 | 1.2 |
| Site 22 | Philips Medical Systems | Achieva | 3 | 6.9 | 3.2 | 8 | 1.2 |
| Site 27 | Siemens | TrioTim | 3 | 1900.0 | 2.3 | 9 | 1.0 |
| Site 52 | Siemens | TrioTim | 3 | 2300.0 | 3.0 | 9 | 1.0 |

**Supplementary Table 2**. Detailed features extracted from each region-of-interest.

| Type | Measure |
| --- | --- |
| First order | *Interquartile Range* |
|  | *Skewness* |
|  | *Uniformity* |
|  | *Median* |
|  | *Energy* |
|  | *Robust Mean Absolute Deviation* |
|  | *Total Energy* |
|  | *Maximum* |
|  | *Root Mean Squared* |
|  | *90° Percentile* |
|  | *Minimum* |
|  | *Entropy* |
|  | *Range* |
|  | *Variance* |
|  | *10° Percentile* |
|  | *Kurtosis* |
|  | *Mean* |
|  | *Mean Absolute Deviation* |
| GLDM | *Gray Level Variance* |
| (Gray Level Dependence matrix) | *High Gray Level Emphasis* |
|  | *Dependence Entropy* |
|  | *Dependence Non Uniformity* |
|  | *Gray Level Non Uniformity* |
|  | *Small Dependence Emphasis* |
|  | *Small Dependence High Gray Level Emphasis* |
|  | *Dependence Non Uniformity Normalized* |
|  | *Large Dependence Emphasis* |
|  | *Large Dependence Low Gray Level Emphasis* |
|  | *Dependence Variance* |
|  | *Large Dependence High Gray Level Emphasis* |
|  | *Small Dependence Low Gray Level Emphasis* |
|  | *Low Gray Level Emphasis* |
| GLCM | *Joint Average* |
| (Gray level Cooccurence matrix) | *Sum Average* |
|  | *Joint Entropy* |
|  | *Cluster Shade* |
|  | *Maximum Probability* |
|  | *Inverse Difference Moment Normalized* |
|  | *Joint Energy* |
|  | *Contrast* |
|  | *Difference Entropy* |
|  | *Inverse Variance* |
|  | *Difference Variance* |
|  | *Inverse Difference Normalized* |
|  | *Inverse Difference Moment* |
|  | *Correlation* |
|  | *Autocorrelation* |
|  | *Sum Entropy* |
|  | *Maximal Correlation Coefficient* |
|  | *Sum Squared* |
|  | *Cluster Prominence* |
|  | *Informational Measure of Correlation 2* |
|  | *Informational Measure of Correlation 1* |
|  | *Difference Average* |
|  | *Inverse Difference* |
|  | *Cluster Tendency* |
| GLRLM | *Short Run Low Gray Level Emphasis* |
| (gray level run length matrix) | *Gray Level Variance* |
|  | *Low Gray Level Non Uniformity Normalized* |
|  | *Gray Level Non Uniformity Normalized* |
|  | *Run Variance* |
|  | *Gray Level Non Uniformity* |
|  | *Long Run Emphasis* |
|  | *Short Run High Gray Level Emphasis* |
|  | *Run Length Non Uniformity* |
|  | *Short Run Non Uniformity* |
|  | *Long Run High Gray Level Emphasis* |
|  | *Run Percentage* |
|  | *Long Run Low Gray Level Emphasis* |
|  | *Run Entropy* |
|  | *High Gray Level Run Emphasis* |
|  | *Run Length Non Uniformity Normalized* |
| GLSZM | *Gray Level Variance* |
| (gray level size zone matrix) | *Zone Variance* |
|  | *Gray Level Non Uniformity Normalized* |
|  | *Size Zone Non Uniformity Normalized* |
|  | *Size Zone Non Uniformity* |
|  | *Gray Level Non Uniformity* |
|  | *Large Area Emphasis* |
|  | *Small Area High Gray Level Emphasis* |
|  | *Zone Percentage* |
|  | *Large Area Low Gray Level Emphasis* |
|  | *Large Area High Gray Level Emphasis* |
|  | *High Gray Level Zone Emphasis* |
|  | *Small Area Emphasis* |
|  | *Low Gray Level Zone Emphasis* |
|  | *Zone Entropy* |
|  | *Small Area Low Gray Level Emphasis* |
